# DeepHistoClass: A Novel Strategy for Confident Classification of Immunohistochemistry Images Using Deep Learning

**DOI:** 10.1016/j.mcpro.2021.100140

**Published:** 2021-08-21

**Authors:** Biraja Ghoshal, Feria Hikmet, Charles Pineau, Allan Tucker, Cecilia Lindskog

**Affiliations:** 1 Department of Computer Science, Brunel University London, Uxbridge, United Kingdom; 2 Rudbeck Laboratory, Department of Immunology, Genetics and Pathology, Uppsala University, Uppsala, Sweden; 3 Inserm, EHESP, Irset (Institut de recherche en santé, environnement et travail), UMR_S 1085, Univ Rennes, Rennes Cedex, France; 4 Protim, Univ Rennes, Rennes Cedex, France

**Keywords:** testis, immunohistochemistry, artificial intelligence, machine learning, histology, AI, artificial intelligence, AUC, area under the curve, BNN, Bayesian neural network, CNN, convolutional neural network, CPPD, class predictive probability distance, DHC, DeepHistoClass, DNN, deep neural network, FFPE, formalin-fixed, paraffin-embedded, HBNet, hybrid Bayesian neural network, HOG, histogram of oriented gradient, HPA, Human Protein Atlas, IHC, immunohistochemistry, mAP, mean-average precision, MC, Monte Carlo, MCC, Matthews correlation coefficient, MCMC, Markov chain MC, ROC, receiver operating characteristic, RT, room temperature, TMA, tissue microarray, VGG, Visual Geometry Group

## Abstract

A multitude of efforts worldwide aim to create a single-cell reference map of the human body, for fundamental understanding of human health, molecular medicine, and targeted treatment. Antibody-based proteomics using immunohistochemistry (IHC) has proven to be an excellent technology for integration with large-scale single-cell transcriptomics datasets. The golden standard for evaluation of IHC staining patterns is manual annotation, which is expensive and may lead to subjective errors. Artificial intelligence holds much promise for efficient and accurate pattern recognition, but confidence in prediction needs to be addressed. Here, the aim was to present a reliable and comprehensive framework for automated annotation of IHC images. We developed a multilabel classification of 7848 complex IHC images of human testis corresponding to 2794 unique proteins, generated as part of the Human Protein Atlas (HPA) project. Manual annotation data for eight different cell types was generated as a basis for training and testing a proposed Hybrid Bayesian Neural Network. By combining the deep learning model with a novel uncertainty metric, DeepHistoClass (DHC) Confidence Score, the average diagnostic performance improved from 86.9% to 96.3%. This metric not only reveals which images are reliably classified by the model, but can also be utilized for identification of manual annotation errors. The proposed streamlined workflow can be developed further for other tissue types in health and disease and has important implications for digital pathology initiatives or large-scale protein mapping efforts such as the HPA project.

## Highlights

• A novel method for automated annotation of immunohistochemistry images.
• Introduction of an uncertainty metric, the DeepHistoClass (DHC) confidence score.
• Increased accuracy of automated image predictions.
• Identification of manual annotation errors.

## Graphical Abstract

**Figure:**
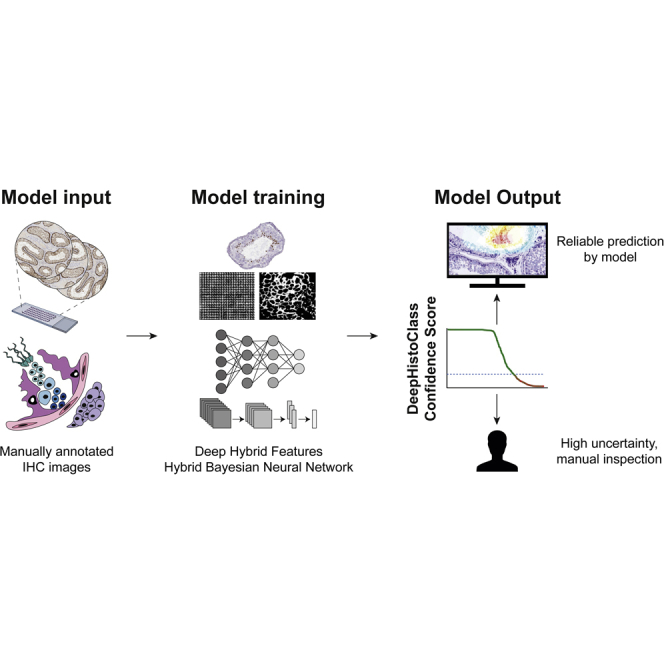

## In Brief

A novel method for automated annotation of immunohistochemistry images, combining the predictions with an uncertainty metric, the DeepHistoClass (DHC) confidence score. This metric not only reveals which images are reliably classified by the model, but can also be utilized for identification of manual annotation errors. The proposed streamlined workflow can be developed further for other tissue types in health and disease and has important implications for digital pathology initiatives or large-scale protein mapping efforts such as the HPA project.

Human physiology depends on complex processes built on intercellular interactions and cell-type-specific functions unique to each tissue and organ. To fully understand the underlying mechanisms of disease, it is necessary to study tissue architecture and molecular constituents with a single-cell resolution. In the field of transcriptomics, dramatic improvements have been made in the single-cell RNA-seq (scRNA-seq) technology, which is a powerful approach due to its excellence in studying mRNAs in smaller subsets of cells that would fall below detection limits when mixed with other cell types in complex tissues samples (1). One major initiative taking advantage of this new technology is the Human Cell Atlas consortium (www.humancellatlas.org). While transcriptomics has the advantage of quantitative measurements and low abundance detection, it is important to note that validation at the protein level is necessary to understand the role in health and disease, as proteomics constitutes the functional representation of the genome. This has recently been shown for expression of the SARS-CoV-2 receptor ACE2, where low abundant measurements based on transcriptomics do not fully reveal the exact localization in tissues unless complemented with proteomics approaches (2).

The standard method for visualizing proteins with a single-cell resolution is antibody-based proteomics and immunohistochemistry (IHC), which allows for studying the protein localization in histologically intact tissue samples. This not only allows for determining the localization in different compartments at a tissue, cellular, and subcellular level, but also provides important information in the context of neighboring cells. IHC thus constitutes an excellent method for direct validation of cell-type-specific expression patterns identified by scRNA-seq. The largest initiative for mapping the human proteome using IHC is the Human Protein Atlas (HPA) project (3, 4, 5, 6, 7), covering all major normal tissues and organs, as well as the most common forms of cancer. The open-access database visualizes the expression of >80% of all human proteins in >10 million high-resolution images, constituting an excellent resource for comparison of cell-type-specific expression patterns identified with large-scale transcriptomics approaches, which has recently been shown in the new Single Cell Type Atlas www.proteinatlas.org/humanproteome/celltype (8).

Despite the IHC technology having been used for decades and is a standard method in clinical pathology, the main approach for evaluation of IHC staining patterns is still a rather subjective manual assessment. A manual observer has the advantage of identifying technical staining errors or artifacts, but it is both time-consuming and costly. Additionally, manual annotation is error-prone and poorly reproducible, as it may lead to fatigue or mislabeling of images due to lack of experience in detecting the correct cell types or structures or technological challenges related to staining intensity or identification of small objects. Manual annotation is commonly faced with two types of errors, i) false negatives where true positive staining is missed or neglected, and ii) false positives where lack of protein expression is falsely interpreted as positive. Histological samples consist of a mixture of different cell types that can be challenging to distinguish even by a trained eye, and setting a manual threshold of what is regarded as negative/positive is tedious and highly difficult. This leads to challenges in large-scale approaches aiming at aligning IHC datasets with data generated by other quantitative methods, such as scRNA-seq.

To increase accuracy and speed up the process of manual interpretation, the application of Artificial Intelligence (AI) in the evaluation of medical images has received increased attention both in research and diagnostics (9, 10, 11, 12, 13). AI-driven and deep learning approaches hold much promise for efficient and accurate pattern recognition of histological images, and there have been several efforts based on IHC images. Most of these previous studies using IHC in machine learning however focused on a smaller number of markers, often well-known biomarkers. These markers were either used to train the algorithm recognizing and measuring the presence of certain cell types within the tissues (14) or to quantify the number of cells positive for a certain marker (15). No previous study has addressed the challenge presented here, training an AI model that distinguishes the cell-type-specific protein expression pattern in human IHC samples, applicable to stainings from any type of protein (16, 17).

One of the challenges when implementing AI models for automated annotation of IHC is that IHC images typically consist of a complex mixture of multiple cell types of various shapes and sizes that can express a protein in different combinations. Additionally, a protein may not only be expressed in certain cell types, but could also be localized to different subcellular compartments, *e.g.*, cytoplasm or nucleus, or be expressed at different levels. As a result, training an algorithm to distinguish cell-type-specific localization of proteins based on IHC is a multilabel task. Since each class is not mutually exclusive, both the manual observer and the trained model must consider every possible label separately. Different approaches to address multilabel classification problems have been developed previously (18), but none of these have been applied to IHC images. Another challenge is correctly addressing the accuracy of automated predictions, which is especially important when implementing algorithms in a clinical setting, but also in whole-proteome approaches such as the HPA project to be able to compare results between different proteins at a global, proteome-wide level. Addressing prediction accuracy requires a large dataset of manually annotated images, but also a method to score the confidence in the prediction. Few existing large-scale imaging datasets are labeled in detail at a cell-type-specific level, and methods for addressing prediction accuracy are not currently considered by many state-of-the-art algorithms. Bayesian neural networks (BNNs) learn a distribution with a prior distribution on its weights and are currently considered state-of-the-art for estimating uncertainty in model prediction, thereby constituting an important element when building automated workflows for annotation of histological images, which was shown in a recent pilot study (19).

In the present investigation, the aim was to present a reliable and comprehensive framework for automated annotation of IHC images that addresses prediction accuracy and that can be used for large-scale approaches. As a model system, we focused on one particular organ—the testis—due to its complex histological features with as many as eight different cell types that can be distinguished by the human eye. These cell stages involved in spermatogenesis and sperm maturation require activation and suppression of thousands of genes and proteins, out of which a large proportion has an unknown function (20, 21, 22, 23, 24). As a basis, we included a large set of 7848 human testis histology images, corresponding to IHC stainings of 2794 different proteins, generated as part of the HPA project. The previous standard HPA annotation in two different testicular cell types for these images was replaced by a new manual in-depth characterization in eight different cell types, which formed the basis for model training in the present investigation. Our automated framework was not only built for recognizing IHC staining patterns at a cell-type-specific level in each of these eight cell types, but also addresses uncertainty with a novel metric—DeepHistoClass (DHC) Confidence Score. The DHC Score is cell-type-specific and combines uncertainty with the predictive label probability, thereby revealing which images are reliably classified by the model, but also has the possibility to identify manual annotation errors.

The proposed streamlined workflow for automated annotation of IHC images constitutes an excellent method for large-scale approaches that currently rely on manual annotation. The method has the ability to discard highly uncertain predictions, highlight which images that need to be checked manually, and can identify unfamiliar patterns or manual errors corresponding to outliers in the data distribution. The method has important implications for large-scale protein mapping efforts such as the HPA project or other digital pathology initiatives, to both save time and lead to higher accuracy in exploration of cell-type-specific protein expression patterns in health and disease.

## Experimental Procedures

### Tissues and Protein Profiling

Human tissue samples for IHC analysis in the HPA dataset were collected and handled in accordance with Swedish laws and regulations. Tissues were obtained from the Clinical Pathology department, Uppsala University Hospital, Sweden, and collected within the Uppsala Biobank organization. All samples were anonymized for personal identity by following the approval and advisory report from the Uppsala Ethical Review Board (Ref # 2002-577, 2005-388, 2007-159). Informed consent was obtained from all subjects in the study, and the procedures follow the Declaration of Helsinki. Generation of tissue microarrays (TMAs), IHC staining, and digitization of stained TMA slides were performed essentially as previously described (25). In brief, formalin-fixed, paraffin-embedded (FFPE) tissue blocks were assembled into TMAs based on 1 mm cores from 44 different normal tissue types corresponding to three individuals per tissue, including normal testis samples from adult individuals. TMA blocks were cut in 4 μm sections, dried overnight at room temperature (RT), and baked at 50 °C for at least 12 h. Automated IHC was performed by using Lab Vision Autostainer 480S Module (Thermo Fisher Scientific), as described in detail previously. The stained slides were digitized with ScanScope AT2 (Leica Aperio) using a 20× objective. All digital images corresponding to antibody data that passed HPA quality criteria were made publicly available on www.proteinatlas.org.

An independent image dataset corresponding to 58 different large sections of clinical samples of human testis was acquired from Institut National de la Santé et de la Recherche Médicale (INSERM) in Rennes, France. Samples were collected over >5 years either from patients undergoing therapeutic orchiectomy for metastatic prostate carcinoma, or from HIV-1-negative cadavers at autopsy at Rennes University Hospital. The protocol for orchiectomy was approved by the Ethical Committee of Rennes, France (authorization n°DC-2010− 1155, June 15, 2011), and written informed consent was obtained from all donors. IHC stainings corresponded to 31 different proteins using HPA antibodies. All stained sections were digitized with a Hamamatsu slide scanner using a 20× objective. Each of the digital images was cropped into multiple images of 3000 × 3000 pixels, to equal the size of the TMA cores in the HPA dataset, and thereby comprising 1218 images used as an independent dataset.

### Experimental Design and Statistical Rationale

We used a BNN-based approach to detect cell-type-specific protein expression from multilabel IHC images. High-resolution digital images of IHC stained testis TMA cores corresponding to 512 testis elevated proteins (24), publicly available on the HPA version 18 (v18.proteinatlas.org), were downloaded along with images from 2282 proteins published in version 19 (v19.proteinatlas.org) that previously had been manually annotated as showing IHC staining of moderate intensity in at least a subset of cells in testis. All proteins were analyzed with at least one antibody that was approved according to HPA criteria for antibody validation. For most of the proteins, three different images were available, and the total dataset comprised 7848 images corresponding to 2794 unique human proteins. Each antibody staining was manually reannotated in eight different testicular cell types, including five germ cell types (spermatogonia, preleptotene spermatocytes, pachytene spermatocytes, round/early spermatids, and elongated/late spermatids), and three somatic cell types (Sertoli cells, Leydig cells. and peritubular cells). The annotation considered staining intensity (negative, weak, moderate, strong) and subcellular localization (cytoplasmic, nuclear, membranous, or a combination of those). The entire dataset was divided into three sets: a training set of 5411 images, a validation set of 1063 images, and a test set of 1374 images. The three sets represent how the entire dataset was divided into work batches as part of the manual annotation workflow, where the validation set corresponding to 1063 images was the original dataset published previously (24). This dataset was manually annotated by one observer and then quality controller by two observers including an expert in testis histology, thereby most likely representing a dataset with little risk of manual errors. The training set, which constituted the largest dataset of 5411 images, was manually annotated by one observer, but not yet quality controlled. Finally, the test set of 1374 images was manually annotated by one observer and quality controlled by one more junior independent observer, but this may not be sufficient to identify all manual errors.

The independent dataset of 1218 images acquired from another laboratory was manually annotated by one observer based on staining intensity and subcellular localization, in the same manner as the training set.

### The Hybrid Bayesian Neural Network (HBNet)

For decades, hand-crafted image features such as Histogram of Oriented Gradients (HOG) (26), Haralick (27), and HU Moments (28) have been widely used in computer vision. The extracted handcrafted features reflect the limited aspects of the problem, yielding low model accuracy and performance depending on the characteristics of the images. Recently we have witnessed a breakthrough in Convolutional Neural Networks (CNN) for image classification and localization tasks. CNNs automatically learn features from high-dimensional images. However, it is difficult to describe what features are learned due to the limited interpretability of CNNs. There is little research on combining CNN features with hand-crafted features for classification tasks. It has been demonstrated that handcrafted features help to provide complementary information for CNNs (29, 30). We propose a Hybrid Bayesian Neural Network (HBNet) method that uses a combination of CNN features (31, 32) and handcrafted features extracted from the all images to provide not only its predicted cell-type-specific protein expression levels, but also a measure of uncertainty estimated using variational Drop Weights to calculate our DHC Score.

In this study, we constructed our HBNet, for extracting deep image features, based on a very deep CNN architecture called VGG Net-19 network. VGGNet was proposed by the Visual Geometry Group (VGG) from the University of Oxford. VGGNet-16 beats the GoogLeNet and obtains an 8.8% error rate. The output of the last convolution layer is the CNN feature. We kept the main characteristics of the VGG Net-19 architecture and connected handcrafted features to the end of the CNN feature as input to the fully connected layers. Handcrafted features were extracted separately from the CNN. These handcraft features mainly reflect color, shape, and texture features of the image as complementary to the CNN features. Drop Weights regularization allowed us to apply variational inference during test time to achieve improved performance. We applied Drop Weights followed by a sigmoid activated layer to the network in the fully connected layer as an approximation to the Gaussian Process (GP), to cast it as approximate Bayesian inference for the meaningful estimation of model uncertainty.

The original JPEG images of 3000 × 3000 pixels were resized to 1024 × 1024 pixels using a bicubic interpolation over a 4 × 4 pixel neighborhood. The handcrafted approaches used were HOG (26), Haralick (27), and HU Moments (28). HOG was applied to all images equally, with eight orientation bins, 8 × 8 pixels forming a single cell, and those cells organized in 8 × 8 formation to form a block. This feature vector containing the image descriptions is the input into the feature selection and classification algorithm. A hybrid feature vector increases the dimensionality of image features. Thus, we used the subspace method to reduce the dimensionality of the hybrid feature vector using PCA to classify and estimate uncertainty in classification. We therefore extracted a 3732-component feature vector by using the HU, Haralick, HOG method post PCA, and a 256-component feature vector using the CNN method.

### Model Training

It should be noted that there are many methods to increase the complexity of the neural network architecture, such as different activation and loss functions, hyperparameter optimization, regularization, spatial and channel information, number of hidden layers of architecture, and multipath information processing, likely to increase overfitting and in turn not necessarily guarantee improvement in accuracy. Finding an optimal neural network architecture, which can be found by trial and error, is therefore an active research area.

During the training process, we used “he_uniform” as the default kernel initializer and the Adam optimizer with AMSGrad = True. The base learning rate was 0.000001 and decreased with the number of iterations. The minibatch size was 32 for 250 epochs and the weight decay factor was 0.2 for the reliability of binary cross-entropy loss decreasing. Overfitting was reduced by using Drop Weights with a rate of 0.3, which means that during both training and inference, approximately one-third of all weights were turned off and set to 0. After training, the output of the last convolution layer was the learned CNN feature. We combined the three handcrafted features (HU, Haralick, HOG) with the CNN features and trained only the fully connected layers and the sigmoid layer. A training dataset (5411 images) and a validation dataset (1063 images) were used for model evaluation. We monitored the validation accuracy after every epoch and saved the model with the best accuracy on the validation dataset. All nonlinearities were ReLU except for the sigmoid output layer. The models were trained and evaluated using Keras with a Tensorflow backend.

During test time (1374 images), Drop Weights were active and Monte Carlo (MC) sampling was performed by feeding the input image with 1000 MC samples through the HBNet. This in turn allowed us to apply variational Drop Weights during testing (19). For every tested image, the model provided not only its predicted class but also a measure of uncertainty estimated using variational Drop Weights (see DHC Confidence Score below). In multilabel classification, a misclassification is no longer necessarily right or wrong, since a correct prediction, containing a subset of the actual labels, is considered better than a prediction containing none of them. We have observed that the use of class weighting during model fitting degrades the performance. In this multilabel detection task, there were many labels that could be present—therefore, we did not want to penalize other classes in favor of only one being present to address class imbalance. The cell type labels in multilabel datasets may be correlated and a prediction for a cell type is not mutually exclusive. Therefore, we utilized label correlation information during classification. For the cost function for multilabel classification, we selected the sigmoid function with the addition of binary cross-entropy. A grid search scheme was adopted based on Matthews Correlation Coefficients (MCC) to determine the optimal thresholds for each dimension on the model outcome, which improves the accuracy of the model. This metric is commonly used to assess multilabel classifiers and can naturally handle asymmetry and class imbalance.

### Multilabel Cross-validation

A Multilabel Stratified Shuffle Split cross-validation merge of Multi-label Stratified KFold and Shuffle Split (33) were used for returning stratified, randomized folds for multilabel data using machine learning classifiers. The folds were made by preserving the percentage of samples for each label repeated ten times in the process of tenfold cross-validation, with different randomization in each repetition.

### Approximate Bayesian Neural Network With Drop Weights Variational Inference for Estimating Model Uncertainty

BNNs provide a natural framework for modeling uncertainty. BNN methods are however intractable in computing the posterior of a network’s parameters. The most common approach to estimate uncertainty in deep learning places distributions over each of the network’s weight parameters. There are many methods proposed for quantifying uncertainty or confidence estimates approximated by MC dropout, including Laplace approximation, Markov chain MC (MCMC) methods, stochastic gradient MCMC variants such as Langevin Dynamics, Hamiltonian methods including Multiplicative Normalizing Flows, Stochastic Batch Normalization, Maximum Softmax Probability, Heteroscedastic Classifier, and Learned Confidence Estimates including Deep Ensembles (34).

Given a dataset $X=\left\{x_{1},\;x_{2}\ldots .\;x_{N}\right\}$ $Y=\left\{y_{1},\;y_{2}\ldots .\;y_{N}\right\}$ and the corresponding labels $Y=\left\{y_{1},y_{2}\ldots y_{N}\right\}$ where $X\in \;R^{d}$$x\in \;R^{d}$ is a d-dimensioned input vector and Y∈{1…….C} y∈{1…….K} with $y_{i}\in \left\{1\ldots \ldots K\right\}$ $y_{i}\in \left\{1\ldots \ldots C\right\}$, *C* class label, a set of independent and identically distributed (i.i.d.) training samples size $N\left\{x_{i},y_{i}\right\}$ for i=1toN, the task is to find a function f:X→Y using weights of neural net parameters *w* as close as possible to the original function that has generated the outputs *Y*. The principled predictive distribution of an unknown label $\hat{y}$ of a test input data $\hat{x}$ by marginalizing the parameters:

$$\;P\left(\hat{y}|\hat{x},X,\;Y\right)=\;\int_{w}P\left(\hat{y}|\hat{x},w\right)P\left(w|X,\;Y\right)dw$$

The expectation of $\hat{y}$ is called the predictive mean of the model, and its variance is called the predictive uncertainty.

Unfortunately, finding the posterior distribution P(w|X,Y) is often computationally intractable. Recently, Gal (34) proved that a gradient-based optimization procedure on the dropout neural network is equivalent to a specific variational approximation on an HBNet. Following Gal (34), Ghoshal *et al.* (35) also showed similar results for neural networks with MC Drop Weights (MCDW). The model uncertainty was approximated by averaging stochastic feed forward MC sampling during inference. During test time, the unseen samples were passed through the network before the Softmax predictions were analyzed. Practically, the expectation of $\hat{y}$ is called the predictive mean of the model. The predictive mean $\mu_{pred}$ over the MC iterations is then used as the final prediction on the test sample: where $\mu_{pred}=\;\frac{1}{T}\overset{T}{\underset{i=1}{\sum }}P\left(\hat{y}|\hat{x},w\right)$. For each test sample $\hat{x}$, the class with the largest predictive mean $\mu_{pred}$ is selected as the predictive probabilities.

### DeepHistoClass (DHC) Confidence Score

Based on the input sample, a network can be certain with high or low confidence of its decision, indicated by the predictive posterior distribution. Traditionally, it has been difficult to implement model validation under epistemic uncertainty. Thus, we predicted that epistemic uncertainty could inform model uncertainty. One of the measures of model uncertainty is predictive entropy *H* of the predictive distribution:

$$H\left(\hat{y}|\hat{x},X,\;Y\right)=\sum_{c=1}^{C}P\left(\hat{y}=c|\hat{x},X,\;Y\right)\;log\;P\left(\hat{y}\;=c|\hat{x},X,\;Y\right)$$

where *C* ranges over all class labels. In general, the range of the obtained uncertainty values is dependent on, *e.g.*, the dataset, network architectures, and the number of MC samples. Therefore, we normalized the estimated uncertainty to report our results and facilitate comparison across various sets and configurations. Estimation of entropy from the finite set of data suffers from a severe downward bias when the data is undersampled. Even small biases can result in significant inaccuracies when estimating entropy. We leveraged the plug-in estimate of entropy and the Jackknife resampling method to calculate bias-reduced entropy (19, 36, 37, 38). The entropy was based on maximizing mutual information between the model posterior density function and the prediction density function, approximated as the difference between the entropy of the predictive distribution and the mean entropy of predictions across samples. Test points that maximize mutual information are points over which the model is uncertain on average, but there are model parameters that produce erroneous predictions with high confidence. This is equivalent to points with high variance in the input to the sigmoid layer (the logits). Thus, each stochastic forward pass through the model would have the highest probability assigned to a different class.

Each prediction from our trained model returned a set of labels. We calculated the DHC Score for each label. We employed the maximum class predictive probability distance (CPPD), which is the difference between the probability values of the highest and the second highest predictive probability value as a measure of a representativeness heuristic. The vector of class probabilities $\;{\hat{y}}_{t}=f^{{\hat{w}}_{t}}\left(\hat{x}\right)$ obtained after the *t* the stochastic forward pass is denoted $\left({\hat{y}}_{t}|\hat{x},{\hat{w}}_{t}\right)$, where ${\hat{w}}_{t}\;$ denotes the sampled parameters resulting from Drop Weights. Thus, the class probabilities of estimates are given by $\frac{1}{T}\sum_{i=1}^{T}P\left(\hat{y}|\hat{x},{\hat{w}}_{t}\right)$. We obtain the CPPD:

$$CPPD\;\left(x_{i}\right)\;=argmin\;\left(\frac{1}{T}\sum_{i=1}^{T}P\left({\hat{y}}_{Best}|\hat{x},{\hat{w}}_{t}\right)-\;\frac{1}{T}\sum_{i=1}^{T}P\left({\hat{y}}_{NextBest}|\hat{x},{\hat{w}}_{t}\right)\right)$$

The MCDW estimate of the vector of class probabilities aimed to decompose the source of uncertainty. The main idea was to select samples that were not only highly uncertain but also highly representative. Based on this strategy, we defined the DHC Score as an approximation of semiautomated sample selection as below:

$DHC\;=\;\frac{CPPD\left(x_{i}\right)}{{\hat{H}}_{J}}$, where ${\hat{H}}_{J}\;$ is bias-corrected entropy using the Jackknife method. In practice, DHC ≈1 means that class predictive probability distance and uncertainty are relatively similar. This happens if a) the model has failed to reach a consensus (class membership difference is small) but model uncertainty is low, or b) the models have reached a consensus (class membership difference is large) but model uncertainty is high. DHC > 0 means that uncertainty is much larger than class membership difference. This set of images represents uncertain predictions. DHC --> ∞ means that uncertainty is much smaller than difference. This represents predictions with high confidence.

We ranked all unlabeled samples in ascending order of DHC Score. The formulation for the sample selection measure can be given as $\;x_{DHC}=\;argsort\;\left\{DHC_{x}\right\}\;\left[:\;sample\;size\right]$. The higher the DHC Score, the higher the information content of the corresponding sample images, which should represent certainty in predictions. The DHC Score was used along with the predictive probabilities, to identify and discard images for which specific cell types did not express a particular protein, as well as images that expressed the protein with high confidence.

## Results

### Generation of a Semiautomated Image Annotation Framework

**Fig. 1:**
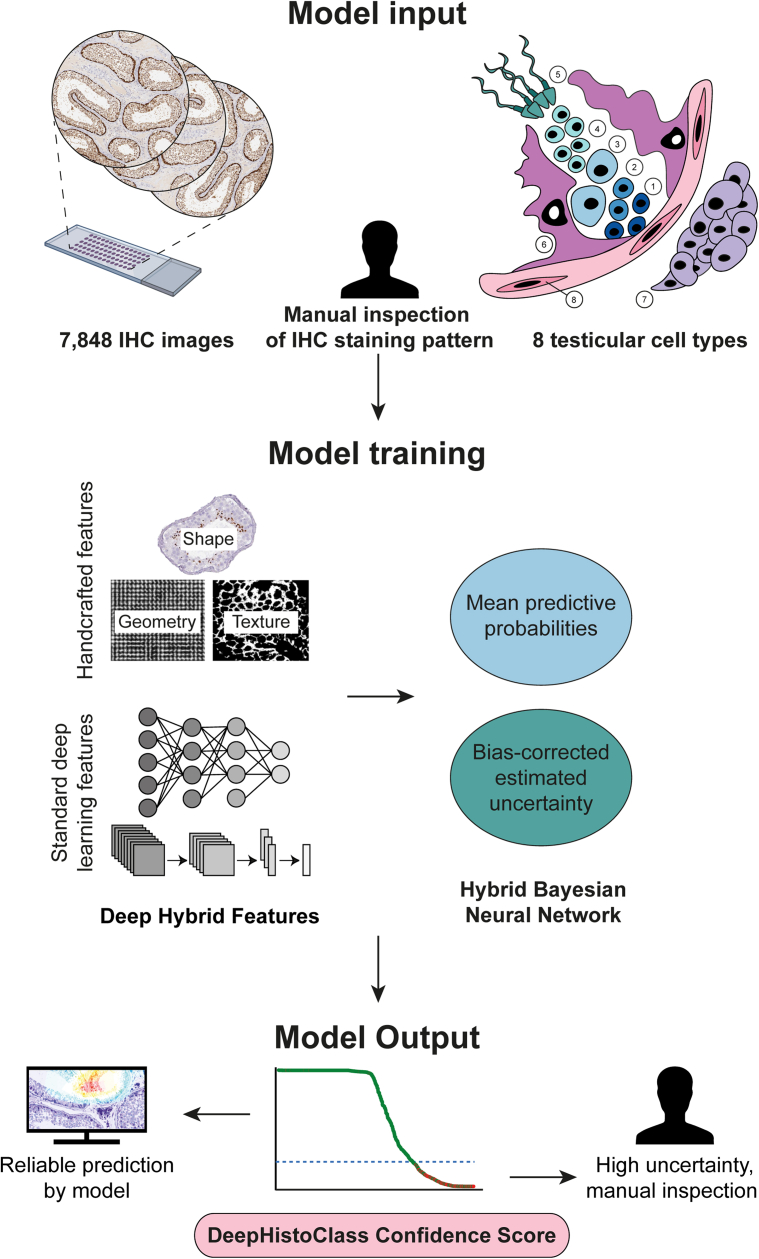
**Overview of the image annotation framework.** A Hybrid Bayesian Neural Network (HBNet) model was trained taking into consideration both handcrafted features and deep learning features. The input IHC high-resolution images consisted of 1 to 3 human testis TMA punch-outs for each antibody comprising a total of 7848 images. For each antibody, eight different cell types were manually inspected with regard to staining intensity (negative, weak, moderate, strong) and subcellular location (cytoplasm, nucleus, membrane); 1: Spermatogonia; 2: Preleptotene spermatocytes; 3: Pachytene spermatocytes; 4: Round/early spermatids; 5: Elongated/late spermatids; 6: Sertoli cells; 7: Leydig cells; 8: Peritubular cells. The manual data was used as a basis for machine learning, combining handcrafted features with standard deep learning features. The mean predictive probability and bias-corrected estimated uncertainty were used for generation of DeepHistoClass (DHC) Confidence Score, which allowed for dividing the images into those that were reliably predicted by the model, and those of high uncertainty that need manual inspection.

A total of 7848 IHC stained high-resolution images of human testis available as part of the HPA project (www.proteinatlas.org), corresponding to 3046 different antibody stainings and 2794 unique proteins, were divided into three different sets: a training set (5411 images), a validation set (1063 images), and a test set (1374 images). All images were annotated manually in five germ cell types (spermatogonia, preleptotene spermatocytes, pachytene spermatocytes, round/early spermatids, and elongated/late spermatids) and three somatic cell types (Sertoli cells, Leydig cells, and peritubular cells), taking into consideration staining intensity (negative, weak, moderate, strong) and subcellular localization of the staining (cytoplasm, nucleus, membrane). This novel refined scoring in eight different cell types formed the basis for a semiautomated image annotation framework, as presented in Figure 1.

### Cell-type-specific Expression Based on Manual Annotation

**Fig. 2:**
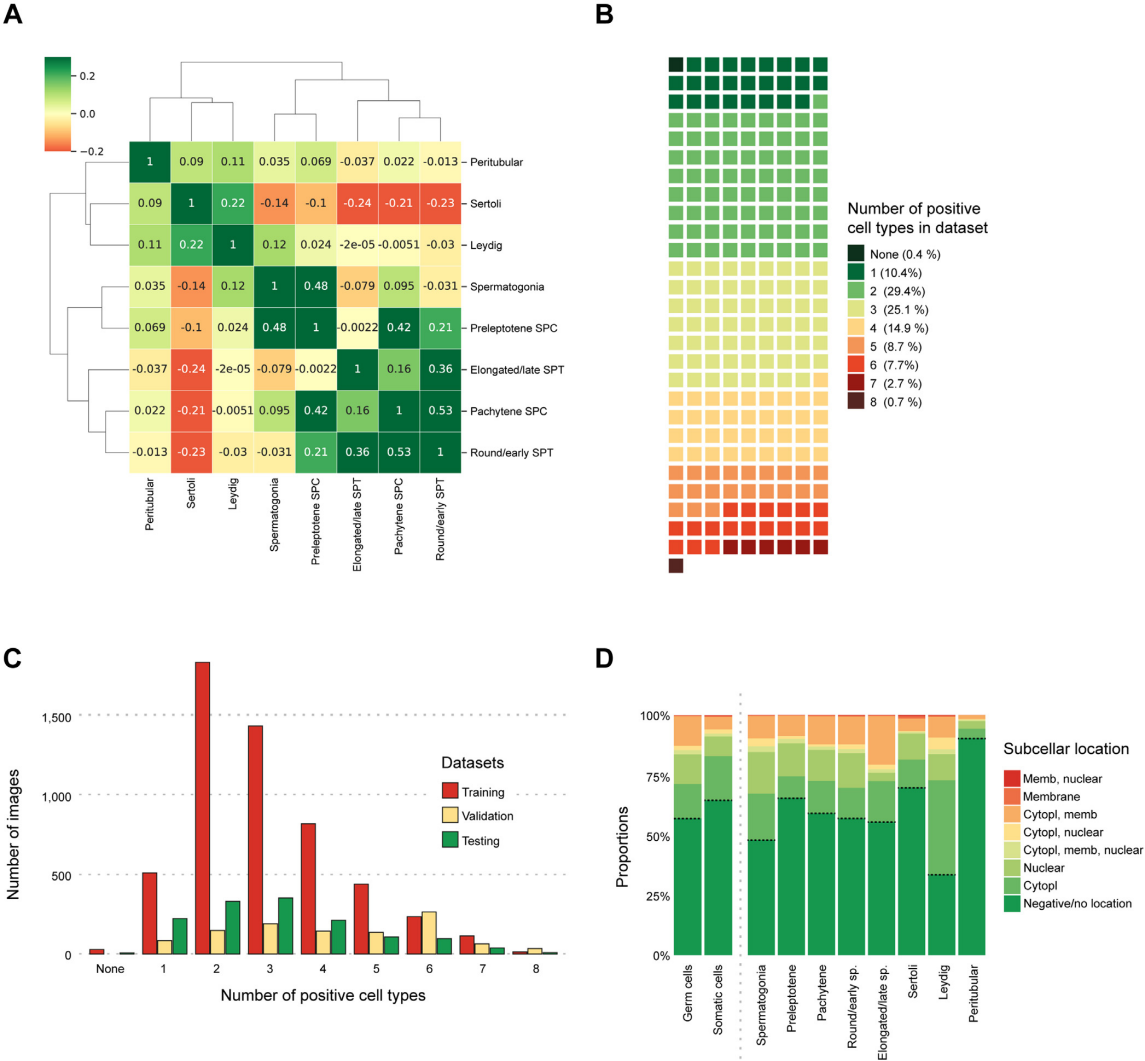
**Input image data distribution based on manual annotation.***A*, heatmap and cluster analysis of testicular cell types. *B*, all 7848 images were grouped based on the number of positive cell types (or lack of positive cell types) and visualized as a waffle distribution plot, which shows that most images contain 2 to 5 positive cell types. In (*C*), the number of positive cell types is visualized separately by each dataset. The training set consisted of 5411 images, validation set 1063 images, and testing set 1374 images. *D*, the distribution of subcellular location (and lack of subcellular location due to no antibody staining) for each cell type in all 7848 images showed that Leydig cells more often showed cytoplasmic staining, while Sertoli cells and peritubular cells had the highest proportion of images that were negative/lacked protein expression in these cell types.

To get an overview of the protein expression pattern across the entire dataset, and determine the relationship between the eight different cell types, pairwise Kendall correlation was used to create a heatmap of the protein expression correlations and the associated clusters (Fig. 2*A*).

The analysis was based on the manual annotation of staining intensity across the entire dataset of 7848 images. As expected, based on functional characteristics (24), there were three main clusters: i) somatic cells (Sertoli cells, Leydig cells, and peritubular cells), ii) premeiotic cells (spermatogonia and preleptotene spermatocytes), and iii) meiotic/postmeiotic cells (pachytene spermatocytes, round/early spermatids, and elongated/late spermatids). Of the 7848 images analyzed, only 815 (10%) showed immunoreactivity in 1 cell type only, while most of the images were positive in 2 to 5 cell types (Fig. 2*B*). In 35 images, the human observer had marked all cell types as negative. When separated, the three different sets showed slightly different proportions of the number of positive cell types (Fig. 2*C*), where the test set consisted of more cell-type-specific images and the validation set contained a higher proportion of images with 5 to 8 cell types that had been labeled (Fig. 2*C*). There were large differences in the presence of different cell type labels (Fig. 2*D*), with Leydig cells being labeled in as many as 5218 (66%) of the images, while peritubular cells represented the most unusual staining pattern, positive in only 755 (10%) of the images. The staining was mostly localized to the cytoplasm, both cytoplasm and the plasma membrane, or the nucleus, but there were clear differences between cell types. Sertoli cells more often showed positivity in the plasma membrane or a combination of nucleus + membrane, in most cases referred to as the nuclear membrane. A majority of the staining observed in Leydig cells was cytoplasmic (Fig. 2*D*).

### Training of Neural Network and Overall Model Performance

The manually annotated images from the training set of 5411 images and the validation set of 1063 images were used for training a HBNet model, exploiting Drop Weights and combining the features from a standard deep neural network (DNN) with handcrafted features. The output of the neural network is an eight-dimensional probability vector, where each dimension indicates how likely each cell type in a given image expresses the protein. The neural network was then applied to the test set of 1374 images, for which the accuracy was evaluated.

Evaluation metrics for multilabel classification performances are different from those used in binary or multiclass classification (39). In multilabel classification, a misclassification is no longer a definite right or wrong, since a correct prediction containing a subset of the actual labels is considered better than a prediction containing none of them. Here, four different metrics were used for evaluating the multilabel classification performance: i) Hamming loss, ii) F1-score, iii) Exact Match ratio, and iv) mean-Average Precision (mAP). Table 1 presents the statistics for each of these metrics for the standard DNN and the proposed HBNet, as well as a host of other state-of-the-art classifiers using our hybrid features. Hamming loss is the most common evaluation metric in multilabel classification, which takes into account both prediction errors (false positives) and missed predictions (false negatives), normalized over the total number of classes and total number of samples analyzed. The smaller the value of Hamming loss (closer to 0), the better the performance of the learning algorithm. F1 score is the harmonic mean of recall and precision, where Macro F1 score calculates the metric independently for each label and then takes an average, and Micro F1 score aggregates the contributions of all labels when calculating the average metric. The Exact Match ratio is the strictest metric, indicating the percentage of all analyzed samples that have all their labels classified correctly. mAP takes into account both the average precision (AP) separately for each label and the average over the class. It provides a measure of quality across recall levels and was shown to be stable and able to distinguish between cell types. The higher the mAP (closer to 100), the better the quality. In the present investigation, there was considerable improvement using HBNet across all metrics used (Table 1). Based on HBNet, the Exact Match ratio showed that 67% of the 1374 images were correctly classified in all eight cell types.

**Table 1:** Overall model performance

Metrics	Neural network (handcrafted features)	Multilabel k nearest neighbours (hybrid features)	Random forest classifier (hybrid features)	Support vector machine (hybrid features)	Hybrid features DNN (%)	Hybrid features BNN HBNet (%)
Hamming Loss	17.0	13.0	15.0	14.0	17.0	13.0
Macro F1 Score	77.0	82.0	77.0	81.0	81.0	84.0
Micro F1 Score	78.0	83.0	79.0	81.0	80.0	84.0
Exact Match ratio	41.0	70.0	47.0	61.0	48.0	67.0
mean-Average Precision (mAP)	70.0	73.0	69.0	72.0	71.0	76.0

Evaluation of classification performance for a Handcrafted features with Neural Network, CNN features Neural Network, Hybrid features Multilabel k Nearest Neighbors, Hybrid features Random Forest Classifier, Hybrid features Support Vector Machine, Hybrid features deep neural network (DNN), and the proposed Hybrid Bayesian Neural network (HBNet), based on five different metrics. The results for each metric are shown as a percent.

### Cell-type-specific Model Performance

**Fig. 3:**
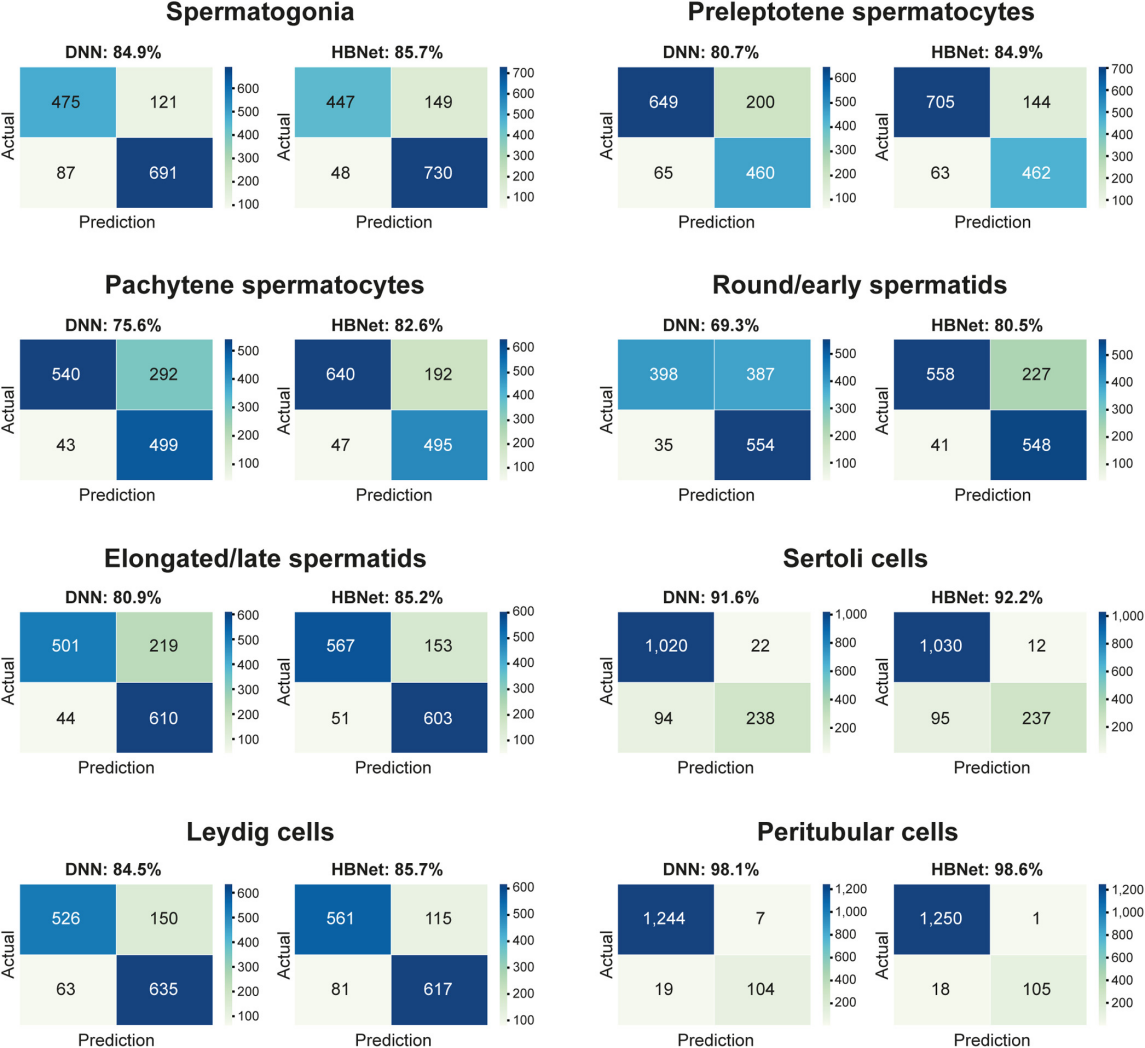
**Confusion matrix for each of the eight testicular cell types based on standard deep neural network (DNN) and hybrid Bayesian neural network (HBNet).** Each quadrant shows the number of images that were true negative (*upper left*), false negative (*upper right*), false positive (*bottom left*), and true positive (*bottom right*), color-coded based on the number of images.

Next, we evaluated the model’s performance on a cell-type-specific level. In Figure 3, a confusion matrix is shown, comparing the output of the neural network with the manual observer, summarizing the false positives and negatives of the DNN and the HBNet for each cell type. For all cell types, HBNet had a higher accuracy than DNN, with >80% overall accuracy, and >90% for Sertoli cells and peritubular cells. The largest difference between DNN and HBNet was seen for pachytene spermatocytes and round/early spermatids, where the accuracy improved from 75.6 to 82.6% and from 69.3 to 80.5%, respectively. HBNet dramatically reduced the number of false negatives compared with DNN, but also showed a decrease in the number of false positives. The total number of false positives (n = 444) across all cell types was lower compared with the number of false negatives (n = 993), indicating that the model performed better at accurately detecting positive labels, but more often differed with the human observer in classifying cell types as negative. This is expected, due to the human observer deliberately neglecting very weak staining patterns that can be considered unspecific or being due to artifacts. The ratios between false positives and false negatives were however opposite for Sertoli cells and peritubular cells, for which false negatives were rare. Positivity in these cell types was not only less common in general (Fig. 2*D*), but also to a larger extent cell-type-specific and not as often showing simultaneous staining in other cell types (Fig. 2*A*). This suggests that positivity in these cell types was mostly considered as specific by the human observer.

### Estimation of Model Certainty

**Fig. 4:**
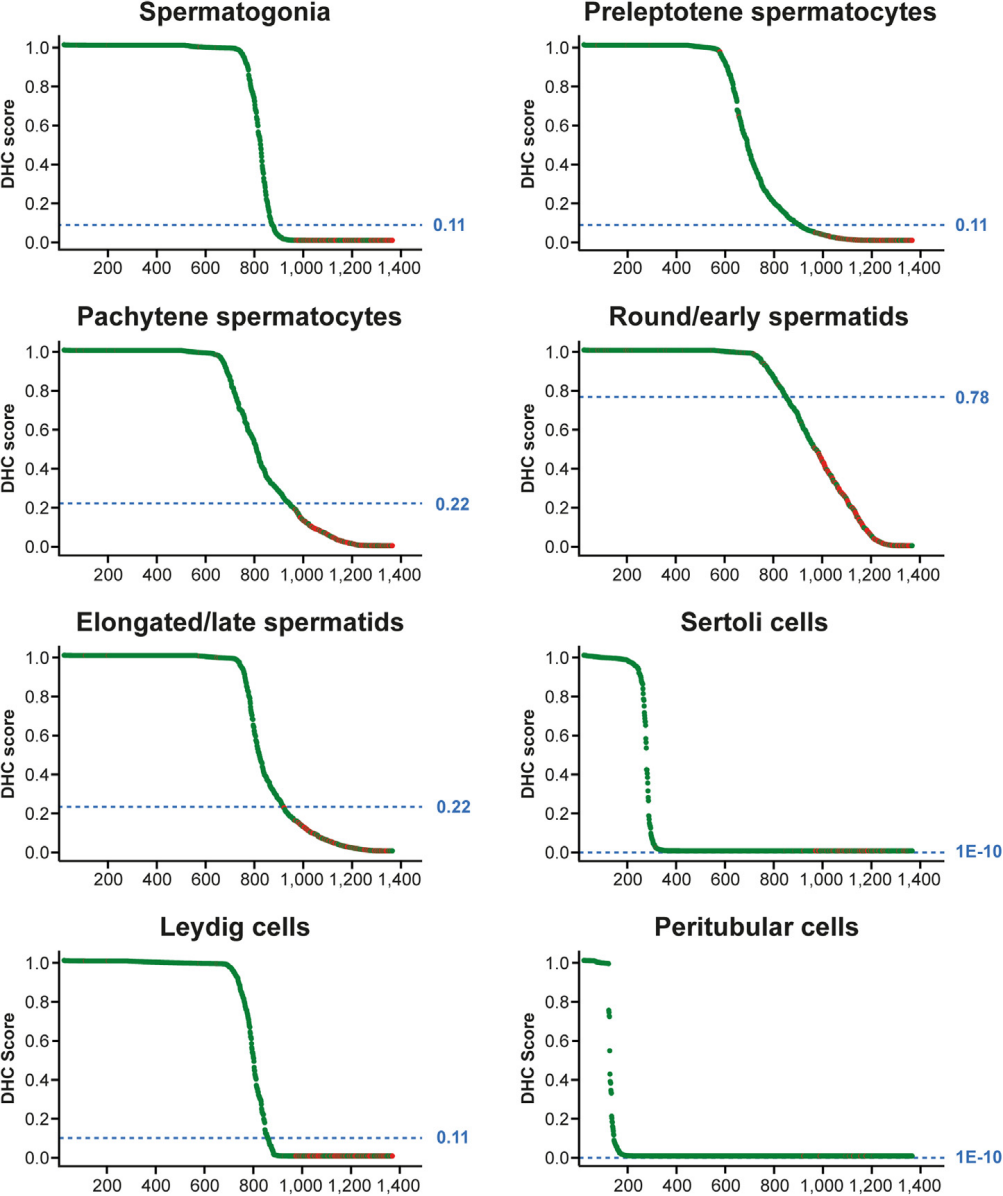
**Confidence maps of all automated predictions for each of the eight cell types.** Each *dot* corresponds to one prediction, with *green* = correct and *red* = incorrect. The predictions were sorted based on their DHC Score, showing the confidence in prediction. The *blue lines* depict the determined cutoff for each cell type where classification is considered too unreliable.

To rank all images based on model confidence over eight cell types, each prediction included an uncertainty measurement, presented as a DHC Score. Supplemental Table S1 shows the predictions per cell type for each of the 1374 images in the test set, along with DHC Score, predictive probability, and manual annotation. The DHC Scores ranged from 0 to 1 for each HBNet prediction over the eight cell types. All predictions were then plotted in confidence maps (Fig. 4), where images for which the model agreed with the human observer, *i.e.*, the cell type was truly positive or truly negative, were marked in green, while images with disagreement between the model and the human observer were marked in red. Images suggested to be misclassified tend to have lower DHC Scores, compared with correctly classified images. The shape of the DHC curves varies for each cell type, and the curves for Sertoli cells and peritubular cells stood out as having a higher proportion of images with low DHC Scores than the other cell types. This is because staining in these cell types was less common (Fig. 2*D*), and cell types classified as lacking staining often have low DHC Scores. The spread of misclassifications determined the cutoff for reliable classification, which was marked as a blue line. Note that this cutoff was set at a DHC Score between 0.0 and 0.11 for all types except pachytene spermatocytes, round/early spermatids, and elongated/late spermatids, for which it was set at 0.22, 0.78, and 0.22, respectively. The protein expression patterns of these three cell types showed a high correlation (Fig. 2*A*), suggesting that many proteins were coexpressed in these cells. Since they were not mutually exclusive, this may explain why the model would have more difficulties to distinguish these cell types from each other. Round/early spermatids are particularly challenging to distinguish manually from the transition into elongated/late spermatids. In the present investigation there were only 67 images with expression restricted to round/early spermatids, while 254 images showed expression specific to elongated/late spermatids and 212 images had expression in both of these two. This likely causes the particularly high DHC Score for round/early spermatids.

**Supplemental Figure S1:**
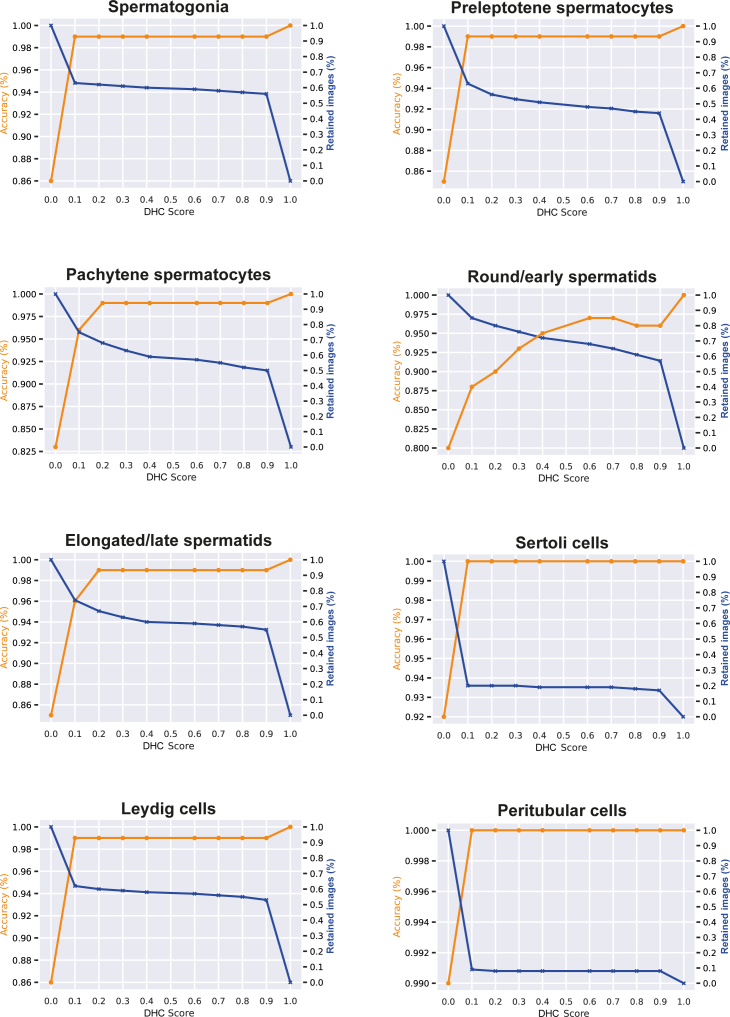


When only considering thresholded samples above the DHC cutoff, including classifications of high reliability, the classification accuracy of the HBNet model was substantially improved and considerably higher than all other classifiers (Table 2). The HBNet DHC-thresholded accuracy was >92% for all cell types except for round/early spermatids, which had an accuracy of 83.5%. For most cell types, approximately 30 to 39% of the images were below the DHC cutoff, except for peritubular cells where only 1.3% of the images were discarded, and Sertoli cells, where none were. Predictions above cutoff can be considered reliably annotated by the model, which means that manual annotation is only needed for on average 28.1% of the predictions. Note that there is a direct trade-off for choice of DHC threshold between accuracy and number of discarded images (supplemental Fig. S1). Also note, accuracy is an orthogonal measure to uncertainty. Similar performance to HBNET may sometimes be obtained with other deterministic classification methods, particularly if they have hybrid features as input, but they do not provide the added value of confidence in their prediction, which enables the identification of images that can be automatically labeled.

**Table 2:** Model performance on a cell-type-specific level

Cell type	Model performance accuracy (%)						Discard tradeoff
	Deep neural network (DNN) with only handcrafted features	Multilabel k nearest neighbours with hybrid features	Random forest classifier with hybrid features	Support vector machine with hybrid features	HBNet (std. dev. Across folds)	HBNet-DHC	HBNet—DHC percentage discarded
Spermatogonia (0.11)	85.9	83.8	80.2	81.2	85.7 (0.24)	99.4	37.2%
Preleptotene spermatocytes (0.11)	74.8	85.5	71.9	73.1	84.9 (0.38)	99.2	37.2%
Pachytene spermatocytes (0.22)	69.9	82.4	72.1	73.3	82.6 (0.24)	99.2	31.7%
Round/early spermatids (0.78)	68.1	79.0	72.8	74.3	80.5 (0.55)	83.5	39.1%
Elongated/late spermatids (0.22)	77.4	79.8	76.9	76.7	85.2 (0.36)	98.7	30.1%
Sertoli cells (1.00E-10)	74.1	86.3	65.9	57.6	92.2 (0.16)	92.2	0.0%
Leydig cells (0.11)	80.4	81.6	80.3	76.2	85.7 (0.34)	99.2	38.3%
Peritubular cells (1.00E-10)	84.3	95.9	67.4	67.9	98.6 (0.09)	98.7	1.3%

The % accuracy for predicting the labels for each cell type is shown for standard deep neural network (DNN) with only handcrafted features, three standard classification approaches including our hybrid features (K-nearest neighbors, random forest, and support vector machines), our hybrid Bayesian neural network (HBNet), and DHC-thresholded HBNet (HBNet—DHC) along with the percentage of discarded images based on low DHC confidence. The standard deviation (std dev.) between each cross-validation fold is included for HBNet to indicate sampling variance.

### Evaluation of Correctly Classified and Misclassified Images

**Fig. 5:**
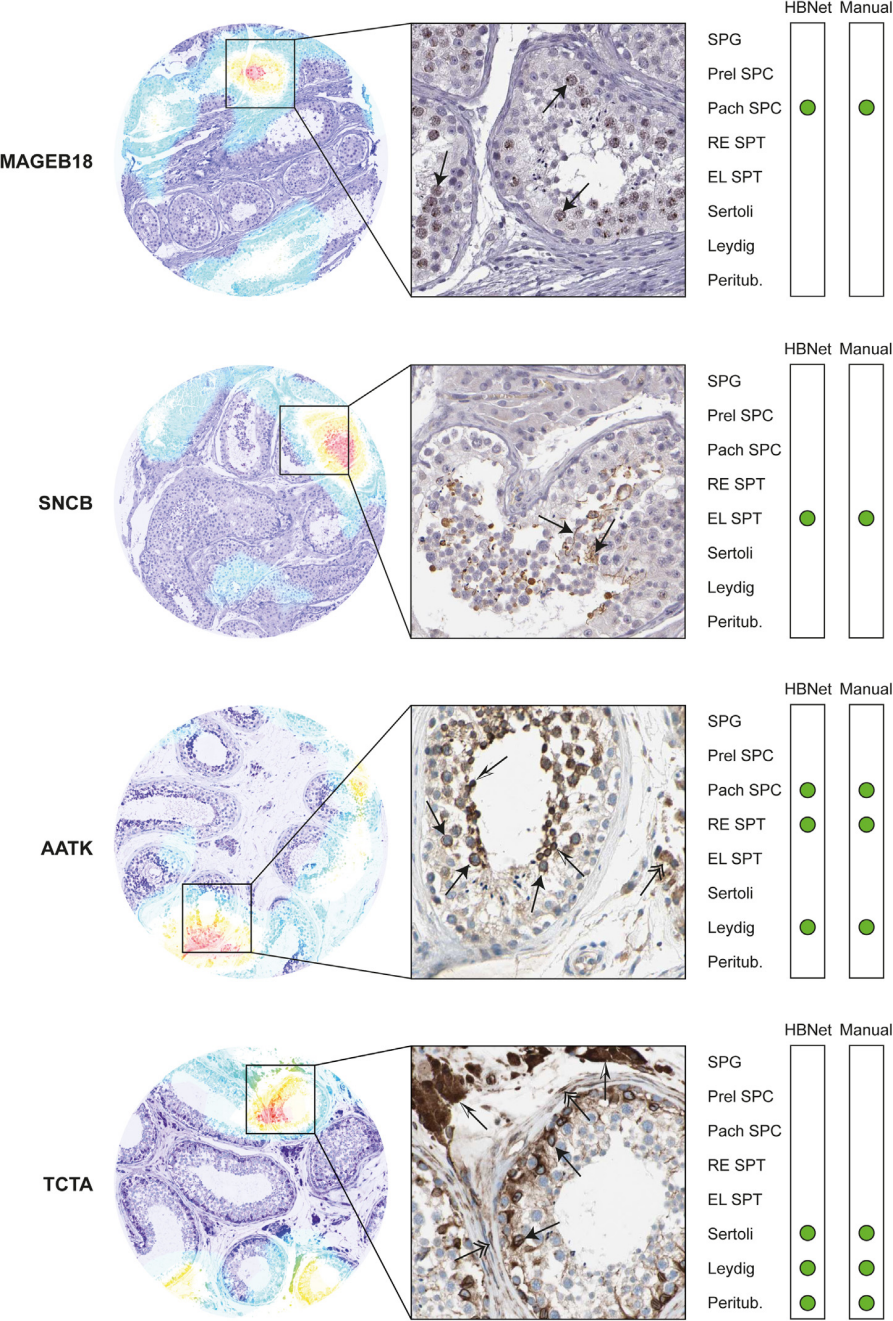
**Examples of correctly classified images.** Heatmaps (*left*), IHC staining patterns (*middle*), with an overview of HBNet prediction and manual annotation of the eight different cell types (*right*). The colors of the heatmaps indicate where the HBNet model focuses on making a labeling decision from purple (no activation) through *blue*, *green*, *yellow*, to *red* (high activation). IHC images show positive staining in *brown* (protein expressed) and counterstaining in *blue* (protein not expressed). Cell type names: Spermatogonia (SPG), preleptotene spermatocytes (Prel SPC), pachytene spermatocytes (Pach SPC), round/early spermatids (RE SPT), elongated/late spermatids (EL SPT), Sertoli cells (Sertoli), Leydig cells (Leydig), and peritubular cells (Peritub.). *Green dots*, correct classification. Melanoma-associated antigen B18 (MAGEB18) and Synuclein beta (SNCB) showed selective expression in 1 cell type only, while Apoptosis-associated tyrosine kinase (AATK) and T cell leukemia translocation altered protein (TCTA) were expressed in several testicular cell types. MAGEB18 showed a speckled nuclear staining pattern in pachytene spermatocytes (*arrows*), with clearly visible nucleoli. SNCB was positive in elongated/late spermatids and sperm flagella (*arrows*), seen in the lumen of seminiferous ducts. AATK displayed cytoplasmic staining in pachytene spermatocytes (*black arrows*), round/early spermatids (*white*/*black arrows*), and Leydig cells (*double-headed arrow*). TCTA showed mainly cytoplasmic staining in Sertoli cells (*arrows*), Leydig cells (*white*/*black arrows*), and peritubular cells (*double-headed arrows*), accompanied with distinct positivity of nuclear membranes in Sertoli cells.

The DHC confidence metric allowed us to identify both correctly classified images and images where the model disagreed with the human observer for one or several cell types. In Figure 5, examples of correctly classified images are provided, *i.e.*, these images were among the 67% that according to the Exact Match Ratio had all eight cell types annotated as either true positive or true negative. The images show that the model performed well both for proteins with distinct and selective staining and for more complex images where the protein was expressed in several cell types of varying intensity and staining patterns. The IHC stained images are presented along with heatmaps (40) highlighting which area of the images that the model focused on for making the labeling decision. For the correctly classified images, it is evident that the model focused on several different areas within the image, including areas where cells were intact and well represented.

**Fig. 6:**
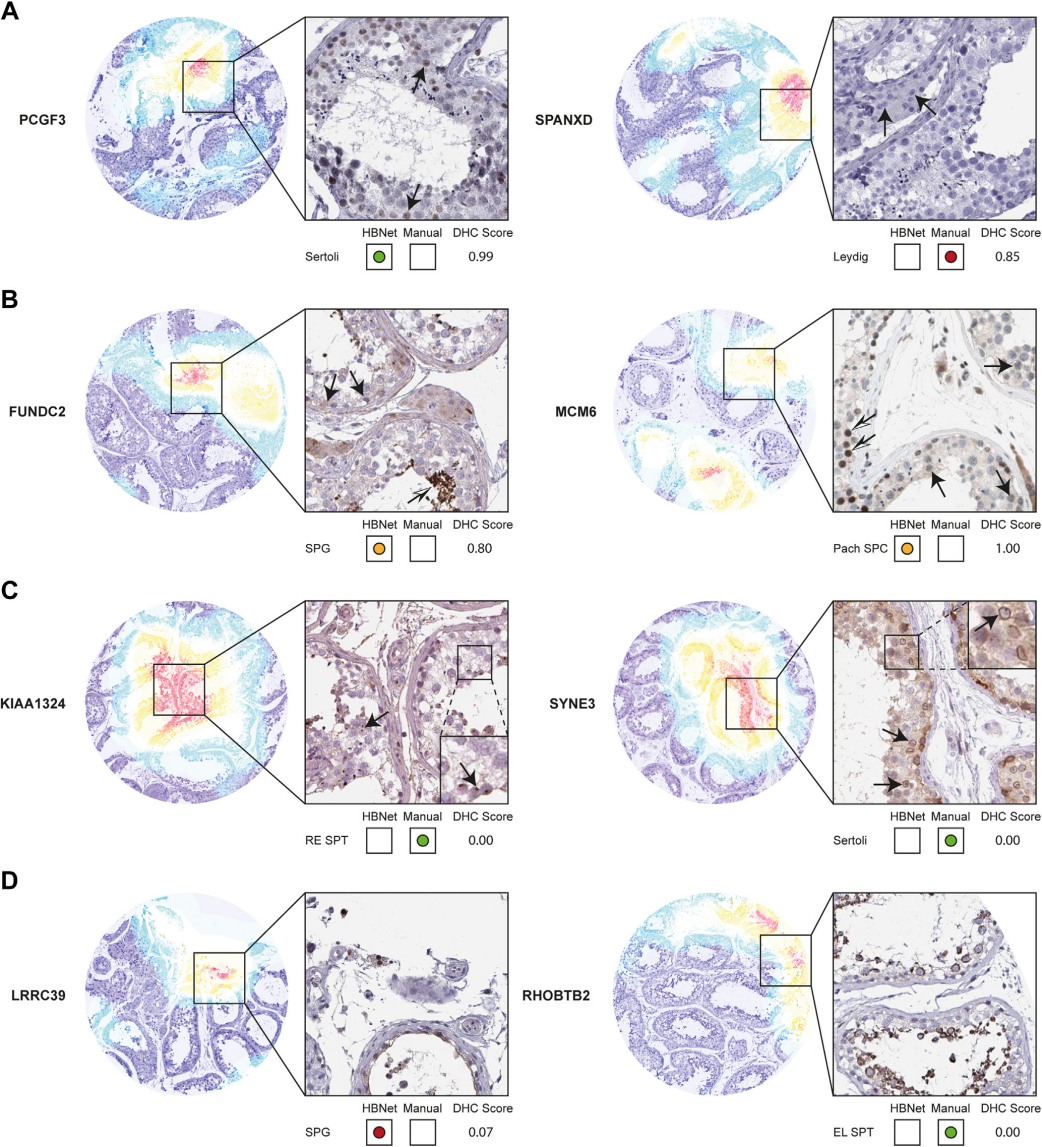
**Examples of misclassified images.** Heatmaps (*left*) and IHC staining patterns (*right*), exemplified by one cell type each where HBNet prediction and manual annotation disagreed. The colors of the heatmaps indicate where the HBNet model focuses on making a labeling decision from *purple* (no activation) through *blue*, *green*, *yellow*, to *red* (high activation). IHC images show positive staining in *brown* (protein expressed) and counterstaining in *blue* (protein not expressed). Cell type names: Spermatogonia (SPG), pachytene spermatocytes (Pach SPC), round/early spermatids (RE SPT), elongated/late spermatids (EL SPT), Sertoli cells (Sertoli), and Leydig cells (Leydig). *Green dots*, correct classification. *Orange dots*, correct classification, but can be considered incorrect based on human knowledge. *Red dots*, incorrect classification. *A*, polycomb group ring finger 3 (PCGF3) and SPANX family member D (SPANXD) represent manual errors. For PCGF3, the manual observer missed Sertoli cells that showed clear nuclear staining (*arrows*), while for SPANXD, Leydig cells had been annotated as positive, despite being completely negative (*arrows*). *B*, FUN14 domain containing 2 (FUNDC2) and Minichromosome maintenance complex component 6 (MCM6) showed staining neglected by the human observer. FUNDC2 displayed weak cytoplasmic positivity in spermatogonia (*arrows*), but due to strong staining in elongated/late spermatids (*white*/*black arrow*), the spermatogonia staining was considered unspecific. Similarly, MCM6 showed weak nuclear staining in pachytene spermatocytes and considered unspecific compared with the strongly positive preleptotene spermatocytes (*white*/*black arrows*). *C*, the uncharacterized protein KIAA1324 and Spectrin repeat containing nuclear envelope family member 3 (SYNE3) were stained in small structures missed by the HBNet prediction. KIAA1324 showed positivity in small perinuclear structures of round/early spermatids most likely representing centrosomes (*arrows*). SYNE3 was stained in nuclear membranes of Sertoli cells (*arrows*). *D*, leucine-rich repeat containing 39 (LRRC39) and Rho related BTB domain containing 2 (RHOBTB2) correspond to images of poor quality. The area for which the HBNet model focused on for prediction of LRRC39 staining only contained unhealthy seminiferous ducts without the correct cell types. Similarly, RHOBTB2 had damaged seminiferous ducts where the cells had been separated from each other and several cell types were missing.

Misclassified predictions included both falsely positive and falsely negative images and could be further divided into cases with high certainty (high DHC Score) and low certainty (low DHC Score). Several misclassified predictions represented clear errors made by the manual observer (Fig. 6*A*). Such misclassifications often had high DHC Scores, and in these cases, the model can be used for identifying manual mistakes. Other misclassified predictions were due to unspecific staining deliberately neglected by the human observer (Fig. 6*B*). Such stainings in need of further protocol optimization were often represented by false-negative predictions with high DHC Scores, indicating that the model performed a correct prediction, but based on experience, the positivity was interpreted as unspecific by the human observer. Some misclassified images corresponded to proteins expressed in small structures including nuclear membranes, nucleoli, or centrosomes (Fig. 6*C*). Such staining patterns are rare and may be particularly challenging for the model to interpret due to limitations in the current pixel resolution. These predictions were often false positives with low DHC Scores. Finally, some misclassified images contained artifacts, such as damaged tissue sections, or sections that contained areas where the testicular samples were not completely healthy (Fig. 6*D*). Such misclassifications, both false positives and false negatives, often had low DHC Scores, and it was evident from the model heatmaps that the labeling decisions were mostly made on areas of the images where not all cell types were clearly represented, or the image/visible cells had poor quality.

### Model Performance Based on Subcellular Localization and Staining Intensity

The manual annotation of the cell-type-specific protein expression did not only take into consideration which cell types were positive, but also in which subcellular organelle the staining was observed. In Table 3, the DHC-thresholded model performance in the test dataset is presented on a subcellular level. Similarly, as in the whole dataset, (Fig. 2*D*), it was clear that some organelles were more common in certain testicular cell types, which may affect the overall accuracy, but it should also be noted that the patterns of different subcellular localizations appear differently in the various cell types based on the cell shape. In total, the best accuracy was found for staining patterns where all subcellular localizations (cytoplasmic, membranous, and nuclear) were present. This is not surprising, as clear outlining of each cell structure increases the likelihood of the model identifying the correct cell types. Sertoli cells had lower accuracy of certain subcellular localizations compared with other cell types. Staining of Sertoli cells is challenging to interpret as these cells have extended cytoplasmic protrusions that occupy the interspaces between the germ cells to provide structural and functional support for their development. Thus, Sertoli cell staining may be difficult to distinguish from other cell types.

**Table 3:** Model performance based on subcellular localization

Cell type	#DHC-thresholded labels/#actual labels with subcellular localization	HBNet—DHC % accuracy (#labels)						
		Cyt	Cyt, Mem	Mem	Nucl	Nucl, Cyt	Nucl, Cyt, Mem	Nucl, Mem
Spermatogonia	518/521	99.5 (204/205)	97.6 (41/42)	100.0 (5/5)	99.5 (201/202)	100.0 (40/40)	100.0 (25/25)	100.0(2/2)
Preleptotene spermatocytes	357/360	100.0 (121/121)	100.0 (30/30)	100.0(2/2)	98.17 (161/164)	100.0(15/15)	100.0(28/28)	0
Pachytene spermatocytes	388/391	99.3 (145/146)	100.0 (66/66)	100.0 (4/4)	98.5 (135/137)	100.0 (9/9)	100.0 (29/29)	0
Round/early spermatids	361/362	99.2 (131/132)	100.0 (57/57)	0	100.0 (147/147)	100.0 (10/10)	100.0 (16/16)	0
Elongated/late spermatids	405/409	98.2 (215/219)	100.0 (83/83)	0	100.0 (67/67)	100.0 (22/22)	100.0 (18/18)	0
Sertoli cells	225/231	97.8 (87/89)	100.0 (31/31)	100.0 (6/6)	95.2 (79/83)	100.0 (1/1)	100.0 (11/11)	100.0 (10/10)
Leydig cells	466/470	98.9 (277/280)	100.0 (71/71)	100.0 (5/5)	100.0 (81/81)	100.0 (25/25)	100.0 (7/7)	0
Peritubular cells	105/120	89.3 (50/56)	100.0 (9/9)	83.7 (46/55)	0	0	0	0
Average all cell types	2825/2864	97.78	99.7	97.28	98.77	100	100	100

In addition to cell-type-specific pattern and subcellular localization of the staining, the human observer also takes into consideration the intensity of the staining. This rather subjective measurement that determines the brown saturation level is considered to represent the amount of protein expression ranging from low levels (weak staining/beige color), through moderate levels (medium brown) to high levels (dark brown/black). As seen in Table 4, it is evident that the DHC-thresholded accuracy did not depend on staining intensity, and there was no significant improvement in predictions performed on distinctly stained cells compared with those that showed more faint positivity.

**Table 4:** Model performance based on staining intensity

Cell type	HBNet—DHC % accuracy (#DHC-thresholded labels/#actual labels)		
	Only weak labels (intensity =1)	Only moderate labels (intensity = 2)	Only strong labels (intensity = 3)
Spermatogonia	100.0 (27/27)	99.3 (142/143)	99.4 (349/351)
Preleptotene spermatocytes	100.0 (49/49)	100.0 (150/150)	98.14 (158/161)
Pachytene spermatocytes	100.0 (70/70)	99.3 (141/142)	98.9 (177/179)
Round/early spermatids	100.0 (53/53)	100.0 (102/102)	99.6 (206/207)
Elongated/late Spermatids	100.0 (41/41)	97.3 (145/149)	100.0 (219/219)
Sertoli cells	98.6 (72/73)	86.1 (31/36)	100.0 (122/122)
Leydig cells	98.9 (172/174)	99.5 (202/203)	100.0 (92/92)
Peritubular cells	100.0 (17/17)	84.5 (49/58)	86.7 (39/45)
Average all cell types	99.7	95.8	97.9

### Validation in an Independent Dataset of Clinical Samples

**Supplemental Figure S2:**
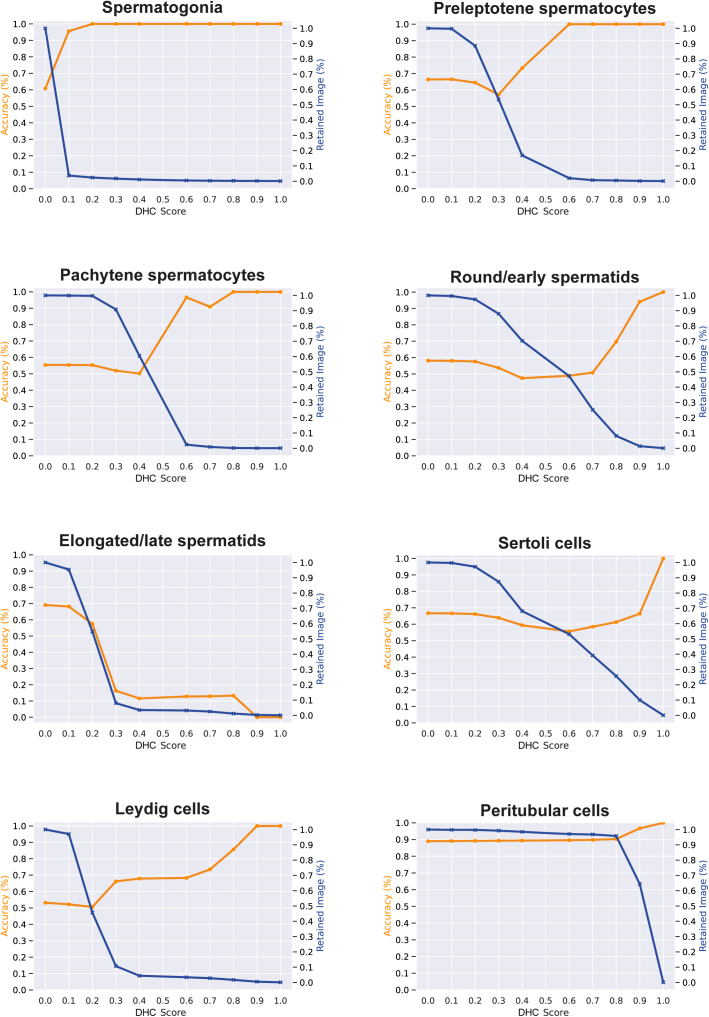


We also explored the use of the same models that were trained on the HPA dataset for classifying images corresponding to clinical samples from a different laboratory. Table 5 documents the results of this independent dataset of 1218 images corresponding to 58 individual samples for the DNN and our proposed HBNet with and without using the DHC Score. The full graphs for trade-off between accuracy and retained images are presented in supplemental Fig. S2, and supplemental Table S2 shows the predictions per cell type for each of the 1218 images in the independent dataset, along with DHC Score and manual annotation. As expected due to the small sample size and significant differences between the laboratories in tissue pretreatment, staining protocol, equipment, and digitization of images, the overall performance was lower with most cell types registering an accuracy of around 60%. When the DHC threshold from the HPA training was used, a general improvement of the accuracies (up to 92%) was observed, but at the expense of discarding a higher proportion of the images as compared with the HPA dataset. Nevertheless, the model did to some degree demonstrate generalizability to images from clinical samples generated from an independent laboratory by successfully identifying a number of images that can be automatically labeled by exploiting the DHC Score.

**Table 5:** Model performance based on independent dataset from another laboratory

Cell types	DNN (Accuracy (%) (std dev.))	HBNet (5000 stochastic feedforward) (std dev.)	HBNet—DHC	HBNet—DHC percentage discarded
Spermatogonia	55.3 (0.78)	60.4 (0.84)	60.4	0%
Preleptotene spermatocytes	61.1 (0.83)	66.2 (1.66)	67.6	36.4%
Pachytene spermatocytes	59.3 (5.4)	55.4 (5.03)	55.4	0%
Round/early spermatids	60.5 (6.54)	58.0 (6.45)	58.2	3.2%
Elongated/late spermatids	61.9 (5.34)	69.1 (8.79)	70.1	1.5%
Sertoli cells	60.8 (3.68)	65.0 (3.73)	66.8	77.2%
Leydig cells	55.2 (3.31)	52.1 (2.45)	55.6	25.6%
Peritubular cells	68.4 (4.69)	89.0 (11.79)	92.7	25.1%

## Discussion

In the evolving era of “big data”, integration of datasets from different omics technologies such as genomics, transcriptomics, and proteomics has shown increasing importance, paving the way for further understanding of the molecular processes involved in health and disease (1). IHC constitutes the standard approach for spatial localization of proteins at a cell-type-specific level. The technology originates from the early 1940s (41) and has emerged as a quick, simple, and cost-effective method applicable to both diagnostic routine, and basic and clinical research. The output of the IHC staining is typically a tissue section manually evaluated under a microscope, but with advances in digital pathology, large-scale digitization of stained sections is becoming more common. Furthermore, novel emerging technologies focusing on highly multiplex efforts, where many proteins are targeted simultaneously in a single tissue section, have received increased attention, further demanding machine learning approaches that can save both time and money and lead to more accurate predictions of IHC images.

Automated algorithms have been widely applied for the recognition of nuclei that can be used for segmentation of specific cells or tissue compartments, *i.e.*, distinguishing between epithelial and stromal cells or between benign and malignant (42, 43, 44, 45, 46), detection of immune cells (47, 48), classification or quantification of certain cell states, such as mitotic cells (49), HER2 positive tumor cells in breast cancer (50), or Ki67 positive proliferative cells (51, 52, 53, 54). Until date, there are however no previous studies suggesting how such frameworks can be implemented for high-throughput annotation of complex tissue samples stained with IHC, applicable to stainings from any type of protein.

Despite impressive reported accuracy, deep learning models tend to require large training sample image sets. While this can be overcome to some degree for many image tasks by using transfer learning (46), there is limited scope for this on IHC images due to the variation in protocols used to process tissue samples across different labs, though this is still a potential area for future work. Deep learning models tend to make overconfident predictions and lack the ability to report “I don’t know” for ambiguous or unknown cases. It is therefore not sufficient to depend on prediction scores alone from deep learning models, but critical to estimate bias-reduced uncertainty as an additional insight to the prediction.

The HPA database based on antibody-based proteomics constitutes the largest and most comprehensive knowledge resource for spatial localization of proteins in organs, tissues, cells, and organelles. The HPA project has characterized >15,000 different proteins across >40 different normal tissues and organs, and 20 types of cancer (3, 4), with the publicly available database www.proteinatlas.org containing >10 million high-resolution images, thereby constituting a major resource for machine learning algorithms. In the present investigation, we focused on generating a novel in-depth annotation dataset based on images of normal testis generated as part of the HPA project, due to the complex architecture of this organ built up by several different cell types, and the unique nature of this tissue harboring a large number of proteins not expressed anywhere else in the human body (11, 22, 23, 55). Selective pressure on most of the genes involved in spermatogenesis implies that different proteins are expressed in certain combinations of these cell types. Some proteins may be expressed in just one subset, while others are more ubiquitously expressed, and the expression of several proteins increases or decreases during differentiation, seen as a gradient in expression in cell states that undergo transformation with differences in size and shape. In addition, Sertoli cells maximize their membrane–membrane contacts with germ cells, resulting in highly entangled tissue. This results in complex IHC images that are very tedious and challenging to interpret manually.

We were careful of the potential impact of image resolution on the performance of the models. Most artificial intelligence or machine learning solutions use significantly downsampled images because of the size of neural networks, which contain millions of parameters. The size and number of images make analysis incredibly demanding, requiring vast computational power. Given the success of deep learning models in image classification, researchers have applied the downsampled techniques used in the ImageNet competitions to medical imaging. Downsampled images are much faster to train deep neural networks. Moreover, lower-resolution images may lead to less overfitting of deep learning models that focus on important high-level features. In the present investigation, a high performance was demonstrated despite using downsampled images, but we may see further improved performance by analyzing the full size images, particularly for staining patterns restricted to certain cellular or subcellular level features.

We here successfully associated deep-learning-based predictions on cell-type-specific protein expression patterns in histological testis sections stained with IHC. Quality metrics that are typically being used in binary classifications or single-label multiclassifications include area under the curve (AUC) or receiver operating characteristics (ROC). In multilabel classification, the predictions constitute a subset of actual class labels, and therefore, the prediction can be fully incorrect, partially correct, or fully correct. As a result, AUC cannot be directly calculated for multilabel classifications but separately computed for each label. Multiple ROC analyses can be carried out through aggregation, but this does not take into account class label imbalance. Here, we assessed multilabel classification using MCC, which is a common metric for analyzing such classifiers. This metric has the attractive property of managing imbalance and asymmetry.

The point predictions were combined with a Confidence Score (DHC), generated by an MC Drop Weights method in conjunction with an approximate BNN with hybrid image features. The proposed HBNet architecture showed outstanding performance in both simple images with clear cell-type-specific staining, and more complex images where several cell types showed positivity of varying intensity and staining patterns. The novel DHC Score adds another level of insight, particularly important for challenging cases where uncertain predictions can be highlighted. The model was tested on an independent dataset of IHC images corresponding to clinical samples from another laboratory, which showed lower overall accuracy. Independent datasets that are generated by different laboratories can be considered the most challenging approach for assessing if a model is fully generalizable, and despite acquiring all images that were digitally available by the other laboratory, it is a limitation that this independent dataset still only corresponded to 58 different samples. Furthermore, these images differed significantly in cell morphology, image quality, color settings during acquisition, as well as the overall brightness and contrast. It is therefore not surprising that the results differed significantly and led to a higher discard rate. Nevertheless, we could still prove the utility of our proposed workflow and achieved high accuracies when filtering the samples that can be automatically labeled based on the uncertainty metric. It should be noted that the proposed HBnet needs to be retrained on data from an individual laboratory before using it to automate labeling in a new setting, rather than trying to generalize between multiple laboratories, unless a universally accepted standardization of IHC staining workflows and digitization of images is introduced. To achieve such a standard is undoubtedly a difficult task, as even stainings generated by the same equipment and protocols may differ between laboratories due to the exact batch or brand of the reagents (56). Additionally, there are several steps in the workflow that can never be controlled for, such as preprocessing and fixation of already existing archived tissue material, making standardization almost impossible. Another possibility for future projects utilizing the proposed workflow is to include images generated by multiple laboratories in the initial training of the model, which would likely improve the overall generalizability.

The unique framework for image annotation allows for dividing the dataset into images that are reliably classified by the model, and images that need to be examined by the manual observer, thereby reducing the manual burden. In addition, our proposed workflow has important implications for identifying images with manual annotation errors and thereby improving the overall accuracy. This is applicable to both research and clinical routine and may replace the otherwise common manual annotation workflow by which one observer first annotates each image, followed by quality control by a second observer, which is the current standard used by the HPA project. It may also be used for teaching purposes in the training of manual observers that have less experience, which saves both time and money as less quality control is needed from experienced personnel.

Weaknesses of an automated algorithm may be related to the fact that manual annotation is not only based on visual examination of staining intensity, but to a large extent also relies on experience, where the manual observer takes into consideration staining protocol, overall image quality, artifacts, and previous literature on the protein being analyzed. Unspecific staining may be neglected by the human observer, especially when accompanied with distinct staining in other structures that more likely represents the true protein expression. Challenges related to tissue processing, IHC staining procedure, and experience in identifying artifacts are however overcome in the presented framework, as uncertain predictions will be highlighted. Our proposed HBNet showed high accuracy for all eight cell types for samples generated by the same laboratory, with increased accuracy after applying a DHC Score threshold. When examining images above and below this threshold, it was evident that many images for which the model faced challenges constituted images expected to be particularly difficult, often due to the reasons described above. Three cell types needed a higher DHC Score threshold for reliable prediction: pachytene spermatocytes, round/early spermatids, and elongated/late spermatids. This is not surprising, as these cells correspond to the most common combination for proteins coexpressed in more than one testicular cell type, as described previously (24).

Previous multilevel classification studies, including a recent Kaggle challenge (57), have used immunofluorescence (IF) images of human cell lines, where antibody staining determined different subcellular localizations of the protein, related to the Subcellular Atlas of the HPA (7, 58). While there are numerous studies focusing on machine learning and IHC, few of these studies aim at distinguishing cell-type-specific protein expression patterns using IHC, a no previous approach can be applied to any type of protein staining (16, 17, 59, 60, 61). In addition to numerous research initiatives, there are several readily available commercial and open-source software supporting IHC images, such as QuPath (15, 62), VisioPharm (63, 64), Halo (65), Aiforia (https://www.aiforia.com/), and Definiens (https://oraclebio.com/). Some of these software require coding abilities, others are fully operational with custom algorithms or built-in easily trained applications by which certain structures are outlined and thresholds are set in a user-friendly interface. Tuning of the software parameters for different images and staining conditions could however be a tedious and time-consuming task in order to make such a workflow applicable to the multilevel task presented here, where each label is represented by a wide range of different staining patterns.

In the present investigation, healthy samples from one particular tissue, and undoubtedly anatomically the most complex in the human body—testis—were used. Based on the encouraging performance of our proposed model for what constitutes a particularly challenging tissue, we believe that the approach is applicable also on other simpler organs with larger structures and less variability in protein expression at the cell-type-specific level. There currently does not exist in any other cell-type-specific dataset as part of the HPA project or any other initiatives with the detailed resolution generated here, but generating more such in-depth characterizations is one of the objectives for future versions of the HPA, as an effort to directly align the protein-based data with single cell level information generated by scRNA-seq. This implies that the suggested workflow can be developed further for other organs in the future, but already now, the method can be used to cover the entire dataset of testis images corresponding to in total >15,000 proteins that have been stained with IHC as part of the HPA project. The workflow can also be used in other large-scale projects focusing on distinguishing between healthy and diseased tissues, widely applicable to, *e.g.*, cancer research but also routine diagnostics, if retrained specifically on datasets from other laboratories. The daily pathology workflow largely depends on manual microscopic evaluation of tissue sections, which may not only lead to a delayed disease diagnosis with potential worsened patient prognosis but also to a false diagnosis (66). Further advances in automated annotation of histological sections are therefore clearly warranted. Many pathology laboratories are now in the transition of starting to become fully digital, and recently the large European initiative BIGPICTURE was formed. This large-scale consortium with 70 million Euros of funding will until the year 2027 create a digital repository of 3 million slides corresponding to a wide range of disease areas. This will open up for new possibilities of linking bioimaging data to clinical parameters with the use of AI, where the proposed workflow that includes addressing of accuracy is an important method to consider.

To summarize, we present a novel method for automated annotation of IHC sections, combining the predictions with an uncertainty metric. The suggested streamlined framework constitutes an important approach for accurate large-scale efforts mapping the human proteome such as the HPA project and holds promise for both research and diagnostics aiming at analyzing the spatiotemporal expression of human proteins in health and disease.

## Data Availability

JPEG files of all 7848 images of the HPA dataset used in the present investigation, as well as the manually annotated protein expression in eight different cell types are available on v20.proteinatlas.org. Manual errors identified as part of this study have been corrected, which means that some of the presented protein expression data on the HPA will differ from the input data used for model training. All images from the independent dataset from another laboratory have been uploaded to the BioStudies repository (https://www.ebi.ac.uk/biostudies) under the accession S-BSST554. All codes are available in GitHub (https://github.com/birajaghoshal/DeepHistoClass).

## Supplemental data

This article contains supplemental data.

## Conflict of interest

The authors declare no competing interests.

## Supplemental Data

Supplemental Table S2

MMC1
