# Supplementary material for: DeepHistoClass: A Novel Strategy for Confident Classification of Immunohistochemistry Images Using Deep Learning
Source: Mol Cell Proteomics. 2021 Aug 21;20:100140. doi: 10.1016/j.mcpro.2021.100140 (PMC8476775; doi:10.1016/j.mcpro.2021.100140)
Supplement: MMC1 [file mmc3.docx]

**SUPPLEMENTAL INFORMATION**

**Supplementary Figure 1. Model accuracy and percentage of discarded images based on DHC threshold in the HPA dataset.** The plots show the accuracy and percentage of discarded images for different values of DHC threshold when applied to the HBNet for each cell type. The plots illustrate the tradeoff between accuracy (orange) and discard rate (blue), where high DHC thresholds result in better accuracy but at the expense of discarding large numbers of images, whereas low thresholds result in the opposite. For most cell types there is a large step increase for the first increments of threshold (between 0.0 and 0.3) followed by far smaller increments (for thresholds over 0.3). This makes choosing a threshold cutoff easy. However, for round/early spermatids the increment is more gradual and constant, resulting in a less clear cut decision point.

**Supplementary Figure 2.** Model accuracy and percentage of discarded images based on DHC threshold in an independent dataset. The plots show the accuracy and percentage of discarded images for different values of DHC threshold for each cell type trained on the HPA dataset and tested on an independent dataset. The plots illustrate the tradeoff between accuracy (orange) and discard rate (blue) where high DHC thresholds result in better accuracy but at the expense of discarding large numbers of images. In comparison with the HPA dataset, the outcomes are more extreme. A small increase in threshold dramatically improves the accuracy, but with the cost of discarding the majority of images.

**Supplementary Table 1. List of images included in the test dataset**. Information on Ensembl ID, antibody ID, gene name, gene description, URLs to HPA JPEG image, as well as model prediction, predictive probability, DHC Score and manual evaluation of staining intensity and subcellular localization for each of the eight analyzed cell types.

**Supplementary Table 2.** List of images included in the independent validation dataset. Information on serial no, image ID, model prediction, predictive probability, DHC Score and manual evaluation of staining intensity and subcellular localization for each of the eight analyzed cell types.
